# Biventricular reverse remodeling and relationship with mitral valve prolapse after transcatheter closure of ASD secundum, a 3D echocardiographic study

**DOI:** 10.34172/jcvtr.2020.03

**Published:** 2019-12-23

**Authors:** Amal El-Sisi, Shaheen Dabour, Aya M Fattouh, Effat Assar, Rasha Naguib, Antoine Fakhry AbdelMassih

**Affiliations:** ^1^Pediatric Cardiology Unit, Pediatrics’ Department, Faculty of Medicine, Cairo University, Cairo, Egypt; ^2^Pediatric Cardiology Unit, Pediatrics’ Department, Faculty of Medicine, Benha University, Benha, Egypt; ^3^Pediatric Cardio-Oncology Department, Children’s Cancer Hospital Egypt (CCHE 57357), Cairo, Egypt

**Keywords:** Reverse Remodeling, MVP, ASD II

## Abstract

***Introduction:*** Mitral valve prolapse (MVP) is the most common anomaly of the mitral valve. Several studies have shown prevalence of MVP in atrial septal defect (ASD) especially secundum types (II). The aims of this study is to show the potential role of 3D echocardiography in improving the diagnosis of MVP and to depict the relationship between reverse remodeling of the right and left ventricles (RV, LV) and MVP after transcatheter closure of ASD II.

***Methods:*** Sixty patients underwent transcatheter closure of ASD II and completed follow up by 2D and 3D echocardiography in Cairo University Children Hospital before the procedure and at 24 hours, 1 and 6 months after the procedure.

***Results:*** 3D echocardiography was more accurate than 2D echocardiography in detecting MVP frequency in ASD II patients (75% vs. 50%). Maximum statistically significant remodeling was detected by 3D echocardiography 1 month after the procedure (RV: LV ratio by 3D echocardiography 1.9±0.03 24 hours after the procedure vs. 1.6±0.03 1 months after the procedure, *P* <0.01) while 2D echocardiography was delayed in detecting biventricular reverse remodeling. 3D derived RV: LV ratio was accurate in detecting MVP status with a sensitivity of 88%.

***Conclusion:*** MVP in ASD II may be related to Biventricular remodeling; 3D echocardiography is accurate in the detection of reverse remodeling as well as MVP in ASD II patients before and after device closure.

## Introduction

Atrial septal defect (ASD) is an abnormal opening in the muscular wall separating the left and right upper chambers (atria) of the heart. ASD accounts for 10% of all congenital heart diseases as it is detected in 1 child per 1500 live births.^1^ The ostium secundum ASD (type II) located in the area of the fossa ovalis is the commonest type of ASD, accounting for almost 75% of cases.^2^

Mitral valve prolapse (MVP) is the commonest anomaly of the mitral valve apparatus. It is defined as the superior systolic displacement of mitral leaflets ≥2 mm above annulus with coaptation point at, or superior to the annular plane. There is increasing evidence that relates the presence of MVP to ASD type II with significant volume overload. The incidence reported for the association between both lesions is 40%-45%.^3^

Several postulations have been made to explain this association with no general consensus reached as of yet. Most studies point towards the distortion in LV geometry as a result of compression by remodeled RV from prolonged volume overload.^4^

Right ventricular eccentric remodeling is a well-known complication of ASD type II, accounting for most of its chronic complications. The dilated RV also compresses the LV leading to its distortion and that of its incorporated mitral valve apparatus leading to MVP.^5^

Right ventricular remodeling, once thought to be a late event after ASD Transcatheter closure, seems to occur earlier than expected; within few hours after ASD closure. However, a major drawback of 2D Echocardiography is that it depends for RV volumes calculations on a geometric assumption which may give the impression of a delayed reverse remodeling after Transcatheter ASD closure.^6^

Cardiac MRI remains the standard method for assessment of RV and LV volumes; however, since 2010 increasing evidence point towards low variability between 3D derived and CMR volumetric assessment of LV and RV.^7^

The aim of this study is to assess the status of MVP after Transcatheter closure of ASD type II and to determine whether or not reverse biventricular remodeling can be correlated with MVP status.

## Materials and Methods

### Study subjects

Sixty-four patients with ASDs indicated for closure according to guidelines^8^ and follow up in Congenital Heart Disease Clinic of Cairo University Children Hospital and Benha University Children Hospital between January 2016 and January 2018 were enrolled for the study. Sixty-two patients had successful device closure and were included in the study. All patients underwent trans-esophageal echocardiography (TEE) to assess their suitability for the device closure. Transcatheter closure of ASD was performed using an appropriately sized Amplatzer-type septal occluder device [St Jude] (also used Occlutech Septal Occluder) in catheter laboratory under TEE guidance. Two patients had lost to follow-up. Sixty patients had completed the study including 1 and 6-months follow-up. Patients with depressed right and/or left ventricular function, severe valvular disease, chronic obstructive pulmonary disease, cardiomyopathy, and residual shunt after percutaneous closure and patients with significant rhythm and conduction disturbances (atrial flutter, atrial fibrillation, atrioventricular [AV] block, and left bundle branch block) were also excluded.

### Study Methods

Transthoracic echocardiography was performed before ASD closure, 24 hours, 1 month and 6 months after ASD closure as follows:

*2D echocardiography* was digitally acquired using a standard protocol. RV/LV ratios were obtained offline from the apical 4 chamber view at end-diastole and end-systole, the basal diameter of RV and LV basal diameters were obtained to calculate the respective end –diastolic and end-systolic RV:LV ratios using the same adopted technique by Akula et al.^9^

*Presence or absence of MVP was assessed according to standard definition*by 2D and 3D echocardiography; three-dimensional billowing height with a cutoff value of 1.0 mm and billowing volume with a cutoff value 1.15 mL was considered diagnostic of MVP by 3D echocardiography.^10^

### 3D echocardiography

Transthoracic examination was carried out with the patient lying in the left lateral recumbent position. Echocardiographic images were stored digitally for offline analysis using Tom Tec software (Tom Tec Imaging Systems, Germany). On the basis of the initial view adjustment and the landmarks, the software automatically calculated the RV and lv end–diastolic and end-systolic volumes which were used to calculate the respective end-diastolic and end-systolic RV:LV volume ratio.^11^

### Statistical Analysis

Data were analyzed using IBM© SPSS© Statistics version 23 (IBM© Corp., Armonk, NY, USA).

Normally distributed numerical variables were presented as mean and SD; inter-group differences were compared using the unpaired *t* test. Paired comparisons for normally distributed numerical data were done using the paired *t* test. Categorical variables were presented as number (%). Receiver-operating characteristic (ROC) curve analysis was used to examine the value of 3D and 2D derived RV: LV ratio in predicting MVP status one month after the procedure. A *P* value <0.05 was considered statistically significant

## Results

Transcatheter closure of ASD type II was performed on 60 patients, with an average hospital stay of 26 ± 2 hours with 0% mortality. All patients had one device implanted; Amplatzer Septal Occluder in 50% of patients, and Occlutech septal Occluder in the rest of patients.

The baseline clinical, demographic and echocardiographic data of the patients were listed in Table 1. Table 2 shows the MVP status and RV: LV ratio by 2D and 3D echocardiography in patients at 4 instances: before the procedure, 24 hours, 1 month and 6 months later. Maximum reduction (15%) in RV: LV ratio by 3D echocardiography has been depicted 1 month after the procedure while the same 15% has been detected by 2D Echocardiography after 6 months of the procedure.

**Table 1:** Clinical, demographic and echocardiographic characteristics of study subjects

**Variables**	**Study subjects** **(n=60)**
Age (y) (Mean±SD)	5±1.2
Male: female ratio	1:2
Heart rate (bpm) (Mean±SD)	124±10
BSA (m^2^) (Mean±SD)	0.89±0.12
Size of ASD II by 2D transthoracic echocardiography	16.6 ± 2.1
Size of ASD II by TTE	16.8±2.2
Percentage of patients with mitral valve prolapse (%) by 3D echocardiography	75%
Percentage of Patients with mitral valve prolapse (%) by 2 D Echocardiography	50%

Abbreviations: ASD II, Ostium secundum atrial septal defect; BPM, beats per minute; BSA, body surface area; n, number; SD, standard deviation; 2D, two-dimensional; 3D, three dimensional.

**Table 2:** Follow up of 2D and 3D derived RV: LV ratios after transcatheter ASD II closure

	**Before closure**	**24 hours after transcatheter closure**	**1 month after transcatheter closure**	**6 months after transcatheter closure**	***P*** **value** ^a^	***P*** **value** ^b^	***P*** **value** ^c^
2D end-systolic RV: LV ratio (Mean ± SD)	2.7±0.09	2.6±0.03	2.6±0.02	2.1±0.05	NS	NS	**<0.01**
2D end-diastolic RV: LV ratio (Mean ± SD)	3.1±0.03	3.1±0.04	3±0.05	3±0.04	NS	NS	NS
3D end-systolic RV:LV ratio (Mean±SD)	2.2±0.05	1.9±0.03	1.6±0.03	1.5±0.02	**0.04**	**<0.01**	NS
3D end-diastolic RV: LV ratio (Mean±SD)	2.5±0.04	2.4±0.04	2.3±0.03	2.1±0.02	NS	NS	**<0.01**
Percentage of patients with MVP by 3D echocardiography, %(n)	75 (45)	75 (45)	45 (27)	40 (24)	NS	<0.01	NS

Abbreviations: ASD II: ostium secundum atrial septal defect; LV, left ventricle; MVP, mitral valve prolapse; n (number); RV, right ventricle; 2D, two-dimensional; 3D, three dimensional. ^a^ *P* value: Comparison between before closure and 24 hours after closure. ^b^ *P* value: Comparison between 24 hours after closure and 1 month after closure. ^c^ *P*value: Comparison between 1 m after closure and 6 months after closure. *P* Value<0.05 was considered statistically significant.

## Discussion

ASD type II is the third commonest congenital heart disease and the commonest congenital heart disease persisting into adulthood. The longstanding left to right shunt through this defect during diastole causes significant RV remodeling. The RV progressively distends causing bowing of the interventricular septum to the left and subsequent shrink of the LV. This mechanism has been regarded as the cause of the common association (45%) between MVP and ASD type II.^12^

In our study subjects, the prevalence of MVP among ASD type II patient was higher than previously reported (75%) when assessed by 3D echocardiography. This may be due to more accurate assessment of prolapsing segments by 3D echocardiography. This may reflect that the prevalence of MVP among ASD type II patients may be underestimated.^13^ This also reflects the need for 3D reconstruction for accurate assessment of MVP status in ASD type II patients.

Transcatheter device closure has become the standard method for closure of ASD type II. Its low profile of postoperative complications gives it the upper hand over closed heart surgery. In our study subjects the 60 patients undergoing the procedure had no reported complications with an average duration of hospital stay of 26±2 hours which is greatly below the expected duration of hospital stay after surgical closure of ASD type II.^14^

There is conflicting data on the duration needed for gradual reverse remodeling of RV in patients after Transcatheter closure of ASD type II. Many papers have focused on this issue from different aspects. These aspects included the hemodynamic status^15^ as well as electrocardiographic,^16,17^ functional,^18-22^ and volumetric reverse remodeling^23-26^; none of which have correlated remodeling to MVP status.

Du et al^25^ suggested that maximum noticeable reverse remodeling occurs 1 month after Transcatheter closure of ASD type II while Santoro et al^23^ and Yılmazer et al^26^ noted earlier reverse remodeling within 24 hours after the procedure.

In our study subjects, 2D Echocardiography failed to show reverse remodeling within 24 hours after device closure, this is contradictory to both Santoro et al^23^ and Yılmazer et al^26^ which both succeeded in showing reduction in RV dimensions by 2D echocardiography. This may be attributed to the larger size of defects in our patients (16.6 ± 2.1) compared to Yılmazer et al (12.24±5). The different size of ASD type II has been reflected as well on the average RV: LV ratio whether by 2D or 3D echocardiography which was pretty higher in our study group.

3D echocardiography at end systole showed an early significant remodeling 24 hours after device closure. However, maximum remodeling has been depicted by 3D echocardiography 1 month after the procedure, while by 2D Echocardiography maximum statistical significance of remodeling has been shown at 6-month post closure. This lag between results of 3D and 2D Echocardiography may be attributable to the tendency of 2D Echocardiography to overestimate ventricular dimensions.^27^

Another striking finding in our study was the discrepancy between the remodeling extent observed at end-systole and that seen at end-diastole. End-diastolic volumes by 3D echocardiography did not show significant reverse remodeling at either 24 hours post-catheterization or after 1 month; to our knowledge our series is the first to examine the reverse remodeling after ASD type II Transcatheter closure at end-systole. An increasing body of evidences in other pathological contexts, point to the fact that reverse remodeling starts at end-systole due to improvement of contractile function of the heart following the abolishment of either volume or pressure load.^28^

To our knowledge this is the first paper to determine the status of MVP following transcatheter ASD closure and to correlate it to Biventricular reverse remodeling. In our patients, significant agreement has been depicted between results of biventricular remodeling as measured by 3D RV: LV ratio and MVP status. None of our patients have shown any change in MVP status 24 hours after the procedure. While 1 month after the procedure 30% of patients showed resolution of MVP, this was also the point at which the maximum change in RV LV ratio by 3D echocardiography has occurred.

## Conclusion

Our study points to a certain delay (1 month) in the occurrence of biventricular reverse remodeling after ASD type II transcatheter closure. Early (after 24 hours) reverse remodeling may be seen in smaller defects with less volume load. It also shows a tight relationship between biventricular reverse remodeling and the improvement of MVP after transcatheter closure of ASD type II, which may mean that the initially observed MVP was attributable to biventricular remodeling. Finally, the study demonstrates that the 3D echocardiography offers a faster and more accurate way in detection of both MVP and biventricular reverse remodeling.

## Competing interests

None.

## Ethical approval

The authors assert that all procedures contributing to this work comply with the ethical standards of the relevant National Egyptian guidelines on human experimentation and with the Helsinki Declaration of 1975, as revised in 2008, and has been approved by the institutional Committees of the Faculty of Medicine, Cairo University.

## Acknowledgement

We want as authors to thank our patients for their priceless trust, but also we want to thank all our students, interns and residents for the viral passion they have. These young colleagues ignite by their enthusiasm our passion and every day seeing them around and working hand in hand with them has the potential ton revive our will to keep up the good work. Last but not least we wanted to send our deepest gratitude to Dr. Rahma A.Menshawey for her valuable advices in English Editing. This outstanding young researcher has substantially helped us.

## References

[1] Bull C, Deanfield J, De Leval M, Stark J, Taylor JF, Macartney FJ (1981). Correction of isolated secundum atrial septal defect in infancy. Arch Dis Child.

[2] Geva T, Martins JD, Wald RM (2014). Atrial septal defects. Lancet.

[3] Suchon E, Podolec P, Plazak W, Tomkiewicz-Pajak L, Pieculewicz M, Mura A (2004). Atrial septal defect associated with mitral valve prolapse--prevalence and clinical significance. Przegl Lek.

[4] Thilén U, Persson S (2006). Closure of atrial septal defect in the adult Cardiac remodeling is an early event. Int J Cardiol.

[5] Ding J, Ma G, Huang Y, Wang C, Zhang X, Zhu J (2009). Right ventricular remodeling after transcatheter closure of atrial septal defect. Echocardiography.

[6] Eroglu E, Cakal SD, Cakal B, Dundar C, Alici G, Ozkan B (2013). Time course of right ventricular remodeling after percutaneous atrial septal defect closure: Assessment of regional deformation properties with two-dimensional strain and strain rate imaging. Echocardiography.

[7] Marsan NA, Westenberg JJM, Roes SD, Van Bommel RJ, Delgado V, Van Der Geest RJ (2011). Three-dimensional echocardiography for the preoperative assessment of patients with left ventricular aneurysm. Ann Thorac Surg.

[8] Baumgartner H, Taylor J (2010). The 2010 version of the ESC guidelines for the management of grown-up adult congenital heart disease are discussed by guidelines task force chairman H Baumgartner. Eur Heart J.

[9] Akula VS, Durgaprasad R, Velam V, Kasala L, Rodda M, Erathi HV (2016). Right Ventricle before and after Atrial Septal Defect Device Closure. Echocardiography.

[10] Chandra S, Salgo IS, Sugeng L, Weinert L, Tsang W, Takeuchi M (2011). Characterization of degenerative mitral valve disease using morphologic analysis of real-time three-dimensional echocardiographic images objective insight into complexity and planning of mitral valve repair. Circ Cardiovasc Imaging.

[11] Vitarelli A, Sardella G, Di Roma A, Capotosto L, De Curtis G, D’orazio S (2012). Assessment of right ventricular function by three-dimensional echocardiography and myocardial strain imaging in adult atrial septal defect before and after percutaneous closure. Int J Cardiovasc Imaging.

[12] Kim SJ (2014). Geometric and functional change of both ventricles after atrial ventricular septal defect closure. J Cardiovasc Ultrasound.

[13] Levine RA, Handschumacher MD, Sanfilippo AJ, Hagege AA, Harrigan P, Marshall JE (1989). Three-dimensional echocardiographic reconstruction of the mitral valve, with implications for the diagnosis of mitral valve prolapse. Circulation.

[14] Siddiqui WT, Usman T, Atiq M, Amanullah MM (2014). Transcatheter Versus Surgical Closure of Atrial Septum Defect: A Debate from a Developing Country. J Cardiovasc Thorac Res.

[15] Schoen SP, Zimmermann T, Kittner T, Braun MU, Fuhrmann J, Schmeisser A (2007). NT-proBNP correlates with right heart haemodynamic parameters and volumes in patients with atrial septal defects. Eur J Heart Fail.

[16] Thilén U, Carlson J, Platonov PG, Olsson SB (2009). Atrial myocardial pathoelectrophysiology in adults with a secundum atrial septal defect is unaffected by closure of the defect A study using high resolution signal-averaged orthogonal P-wave technique. Int J Cardiol.

[17] Santoro G, Pascotto M, Sarubbi B, Bigazzi MC, Calvanese R, Iacono C (2004). Early electrical and geometric changes after percutaneous closure of large atrial septal defect. Am J Cardiol.

[18] Di Salvo G, Drago M, Pacileo G, Carrozza M, Santoro G, Bigazzi MC (2005). Comparison of strain rate imaging for quantitative evaluation of regional left and right ventricular function after surgical versus percutaneous closure of atrial septal defect. Am J Cardiol.

[19] Veldtman GR, Razack V, Siu S, El-Hajj H, Walker F, Webb GD (2001). Right ventricular form and function after percutaneous atrial septal defect device closure. J Am Coll Cardiol.

[20] Ding J, Ma G, Wang C, Huang Y, Zhang X, Zhu J (2009). Acute effect of transcatheter closure on right ventricular function in patients with atrial septal defect assessed by tissue Doppler imaging. Acta Cardiol.

[21] Cheung Y, Lun K, Chau AKT (2004). Doppler tissue imaging analysis of ventricular function after surgical and transcatheter closure of atrial septal defect. Am J Cardiol.

[22] Agha HM, El-Saiedi SA, Shaltout MF, Hamza HS, Nassar HH, Abdel-aziz DM (2015). Incomplete RV Remodeling After Transcatheter ASD Closure in Pediatric Age. Pediatr Cardiol.

[23] Santoro G, Pascotto M, Caputo S, Cerrato F, Cappelli Bigazzi M, Palladino MT (2006). Similar cardiac remodelling after transcatheter atrial septal defect closure in children and young adults. Heart.

[24] Kort HW, Balzer DT, Johnson MC (2001). Resolution of right heart enlargement after closure of secundum atrial septal defect with transcatheter technique. J Am Coll Cardiol.

[25] Du ZD, Cao QL, Koenig P, Heitschmidt M, Hijazi ZM (2001). Speed of normalization of right ventricular volume overload after transcatheter closure of atrial septal defect in children and adults. Am J Cardiol.

[26] Yılmazer MM, Güven B, Vupa-çilengiroğlu Ö, Öner T (2013 (May 2011)). Improvement in cardiac structure and functions early after transcatheter closure of secundum atrial septal defect in children and adolescents. Turk J Pediatr.

[27] Kühl HP, Hanrath P, Franke a (2003). M-mode echocardiography overestimates left ventricular mass in patients with normal left ventricular shape: a comparative study using three-dimensional echocardiography. Eur J Echocardiogr.

[28] Waring AA, Litwin SE (2016). Redefining Reverse Remodeling: Can Echocardiography Refine Our Ability to Assess Response to Heart Failure Treatments?. J Am Coll Cardiol.

